# Effect of *Launaea procumbens* extract on oxidative marker, p53, and CYP 2E1: a randomized control study

**DOI:** 10.3402/fnr.v60.29790

**Published:** 2016-03-03

**Authors:** Rahmat Ali Khan, Muhammad Rashid Khan, Sumaira Sahreen, Huda Mohammad Alkreathy

**Affiliations:** 1 Department of Biotechnology, Faculty of Sciences, University of Science and Technology Bannu, KPK, Pakistan; 2 Department of Biochemistry, Faculty of Biological Sciences, Quaid-i-Azam University, Islamabad, Pakistan; 3 Botanical Sciences Division, Pakistan Museum of Natural History Islamabad, Pakistan; 4 Department of Pharmacology, Faculty of Medicine, King Abdulaziz University, Jeddah, Saudi Arabia

**Keywords:** carbon tetrachloride, *Launaea procumbens*, liver, hepatic antioxidants, CYP 2E1, lipid peroxidation, p53, AgNORs

## Abstract

**Background:**

Ethyl acetate extracts of *Launaea procumbens* is used for the treatment of liver dysfunction as an herbal medicine in Pakistan. In this study, the protective effects of ethyl acetate extracts were evaluated against CCl_4_-induced liver injuries in rat.

**Methods:**

To examine the protective effects against oxidative stress of carbon tetrachloride in rats, 30 male rats were equally divided into 5 groups (6 rats). Among five groups, one was treated with CCl_4_ (3 ml/kg i.p. in olive oil b.w.) twice a week for 4 weeks. Others were orally fed with extracts (100, 200 mg/kg b.w.), with CCl_4_ twice a week for 4 weeks.

**Results:**

Administration of CCl_4_ altered the serum marker enzymes, lipid profile, CYP 2E1, p53 expression, antioxidant enzymes, nuclear organizer regions (AgNORs), and DNA. Supplement of *L. procumbens* ameliorated the effects of CCl_4_, improved CYP 2E1, p53, and increased the activities of antioxidant enzymes while activity of liver marker enzymes (ALP, ALT, AST, g-GT) and contents of lipid per oxidation contents (TBARS), AgNORs, and DNA fragmentation were decreased. Similarly body weight was increased while liver and relative liver weight was decreased with co-administration of various extracts, suggesting that *L. procumbens* effectively protect liver against the CCl_4_-induced oxidative damage in rats.

**Conclusion:**

The hepatoprotective and free radical scavenging effects might be due to the presence of bioactive constituents in the extract.

Hepatitis viral infection, food additives, alcohol, toxins, toxic industrial chemicals, and air and water pollutants are the major risk factors of liver toxicity (1). There is increasing evidence that free radicals and reactive oxygen species (ROS) play a crucial role in the various steps that initiate and regulate the progression of liver diseases independently of the agent in its origin (1). CCl_4_ is a potent environmental hepatotoxin (2) that, in addition to hepatic problems, also causes dysfunction of kidneys, lungs, testes, and brain, as well as generates free radicals in blood (3–5).

CCl_4_ requires bioactivation by phase I cytochrome P450 system to form reactive metabolic trichloromethyl radical (CCl_3_*) and peroxy trichloromethyl radical (*OOCCl_3_). These free radicals can bind with polyunsaturated fatty acid (PUFA) to produce alkoxy (R*) and peroxy radicals (ROO*) that, in turn, generate lipid peroxides that are highly reactive, change enzyme activity, and finally induce injury or necrosis (6). The injuries induced by CCl_4_ result from free radicals through lipid per oxidation of cell membranes. CCl_4_ is known to decrease GSH of phase II enzyme, and reduces antioxidant enzyme and antioxidant substrates to induce oxidative stress that is an important factor in acute and chronic injuries in various tissues (6–8). Free radical of carbon tetrachloride reduces glutathione content and antioxidant activity leads to hepatic injuries (9, 10). CCl_4_ controls the peroxy radicals, thereby depleting the antioxidant enzymes. ROS causes oxidative DNA damages, with the formation of DNA adducts, genetic mutation, strand breakage, and chromosomal alterations (9, 11). Some recent investigation revealed that free radicals induce an increase in the number of nuclear organizer regions (AgNORs); enhance activity of telomerase enzymes activity (9, 12); cause depletion of CYP 2E1 (13); and increase oxo8dG concentration (14). DNA fragmentation causes p53 gene expression; blocks cell cycle, and gives additional time to repair DNA; however, severe DNA damage triggers apoptosis (15).

*Launaea procumbens* was traditionally used in the treatment of rheumatism (16), kidney and liver disorders (17, 18), eyes diseases (19), and as food (20). Nutritional analysis of *L. procumbens* reveals the presence of salicylic acid, vanllic acid, synergic acid, 2-methyl-resercinol, and gallic acid (21). Therefore, the present study was arranged to evaluate the traditional use of ethyl acetate extract of *L. procumbens* versus carbon tetrachloride–induced liver disorders and lipids peroxidation in rats.

## Materials and methods

### Plant collection

*L. procumbens* at maturity was collected from Wah Cantt District Rawalpindi (Pakistan). Plants were identified and a specimen was submitted at Herbarium of Quaid-i-Azam University Islamabad, Pakistan. Aerial parts of plant (leaves, stem, flowers, and seeds) were shade dried at room temperature, chopped, and grinded mechanically to mesh size 1 mm.

### Preparation of plant extract

One-kilogram powder of *L. procumbens* was extracted in 2 L methanol to get crude methanolic extract which was further fractionated to ethyl acetate. The ethyl acetate fraction (LEA) was evaporated under reduced pressure in a rotary evaporator, dried, and stored at 4°C for *in vivo* studies.

### Animals and treatment

A total of 30, six-week-old, male albino rats (180–190 g) were provided by the National Institute of Health Islamabad and were kept in ordinary cages at room temperature of 25±3°C with a 12 h dark/light cycle. They were allowed standard laboratory feed and water. The study protocol was approved by Ethical committee of Quaid-i-Azam University, Islamabad for laboratory animal feed and care.

### Experimental design

To study the antioxidant effects of LEA, male albino rats were equally divided into five groups (six rats). Group 1 was given raw water and free access to food materials. Group II received olive oil intraperitoneally (Monday and Thursday) and DMSO orally (Wednesday and Saturday) at a dose of 3 ml/kg body weight. Group III received CCl_4_ 3 ml/kg intraperitoneally in olive oil (Monday and Thursday). Group IV and V were given orally 100; 200 mg/kg b.w. (in DMSO), (LEA) after 48 h of CCl_4_ treatment (Wednesday and Saturday). After 24 h of the last treatment, all the animals were weighted and sacrificed; their livers were removed, weighted, perfused in ice-cold saline solution, and treated with liquid nitrogen for further analysis.

### Assessment of serum profile

Serum marker enzymes (ALT, AST, ALP, γ-GT) and lipid profile (cholesterol, LDL, HDL, triglyceride) were estimated using standard AMP diagnostic kits (Stattogger Strasse 31b 8045 Graz, Austria). CYP 2E1 and p53 concentration was determined with ELISA kit.

### Assessment of antioxidant status

Liver tissue (70 mg) was homogenized in 10 volumes of 100 mmol KH_2_PO_4_ buffer containing 1 mmol EDTA (pH 7.4) and centrifuged at 12,000× *g* for 30 min at 4^o^C. The supernatant was collected and used for determining antioxidant status as described below using concentration of protein estimated with the method of Lowry et al. (22). Antioxidant status including activity of catalase (23), superoxide dismutase (24), glutathione-*S*-transferase assay (25), glutathione reductase (26), glutathione peroxidase (27), reduced glutathione assay (28), and lipid peroxidation assay (29).

### DNA fragmentation % assay

DNA fragmentation % assay was conducted using the procedure of Wu et al. (30) with some modifications. The liver tissue (50 mg) was homogenized in 10 volumes of a TE solution pH 8.0 (5 mmol Tris-HCl, 20 mmol EDTA) and 0.2% triton X-100. One milliliter aliquot of each sample was centrifuged at 27,000×*g* for 20 min to separate the intact chromatin (pellet B) from the fragmented DNA (supernatant, T). The pellet and supernatant fractions were assayed for DNA content using a freshly prepared DPA (diphenylamine) solution for reaction. Optical density was read at 620 nm with (SmartSpecTM plus Spectrophotometer catalog # 170-2525) a spectrophotometer. The results were expressed as amount of % fragmented DNA by the following formula: % Fragmented DNA=T×100/T+B

### AgNORs count

After weighing small pieces, each liver was fixed for 3–4 h in fixative sera followed by dehydration with ascending grades of alcohol (80, 90, and 100%) and transferred to cedar wood oil. When tissue became clear, all tissues were embedded in paraplast and prepared as blocks for further microtomy. Thin slides (3–4 µm) were prepared with microtome and the wax was removed. After complete removal of wax, the slides were hydrated in decreased ethanol concentration (90, 70, and 50%) and washed in distilled water for 10 min and dried in an oven. After drying, slides were treated with one drop of colloidal solution (2% gelatin and 1% formic acid) and two drops of 50% AgNO_3_ solution onto the slide and incubated at 35°C for about 8–12 min. The progressive staining was followed under a microscope to get golden colored nuclei and brown/black NORs. Then, the slide was washed in distilled water, treated for 1 min with 1% sodium thiosulfate at room temperature to stop the reaction, and washed in tap water. The cells were examined under a light microscope at 100× magnification and the number of AgNORs was counted per cell (31).

### Statistical analysis

To determine the treatment effects one-way analysis of variance was carried out by computer software SPSS 13.0. Level of significance among the various treatments was determined by LSD at 0.05% level of probability.

## Results

### Body weight, liver weights

CCl_4_-induced lipid peroxidation plays a key role in the body weight and the organ weight of rats. Administration of CCl_4_ caused significant diminution (*P<*0.01) in body weight while amplifying AgNORs, tissue, and relative tissue weight as compared with the non-treated normal control rat. Supplementation of 100 mg/kg and 200 mg/kg b.w. LEA significantly restored (*P<*0.01) the weight of body and liver as well as relative weight dose dependently (Table 1).

**Table 1:** Effect of various fractions of *Launaea procumbens* on liver weight, relative liver weight, AgNORs count, and DNA fragmentation % in liver of rat

Treatment	Liver weight (g)	Relative liver weight (g)	% increase in body weight (g)	DNA damages %	AgNORs (NORS/cell)
Control	5.78±0.209†	0.0578±0.00209†	26.0±0.80†	5.17±0.94†	2.167±0.307†
DMSO+olive oil	5.88±0.206†	0.0588±0.00206†	25.9±0.63†	5.00±0.44†	2.667±0.333†
3 ml/kg CCl_4_	6.96±0.194**	0.0696±0.00194**	18.6±0.72**	35.83±0.14**	9.000±0.931**
100 mg/kg LPEE+CCl_4_	6.05±0.0861**†	0.0605±0.00086**†	22.5±0.42†	9.83±0.97*†	7.333±0.667**†
200 mg/kg LPEE+CCl_4_	5.92±0.205†	0.059±0.00205†	24.49±0.54†	7.33±0.67†	5.333±0.882**†
200 mg/kg LPEE alone	5.01±0.32†	0.0523±0.00101†	27.39±0.21†	6.01±0.27†	3.023±0.92†

Mean±SE (*n*=6 number).

*, ** Significance from the control group at *P<*0.05 and *P<*0.01 probability levels.

† Significance from the CCl_4_ group at *P<*0.01 probability level.

### Effects of LEA on lipid profile in rats

The protective effect of LEA on lipid profile is shown in Table 2. Treatment of CCl_4_ significantly increased (*P<*0.01) lipid profile (triglycerides, total cholesterol, LDL cholesterol) while extensively decreasing (*P*<0.01) HDL cholesterol. Reduction of HDL cholesterol was notably (*P*<0.01) enhanced by 100 mg/kg and 200 mg/kg b.w. LEA, while triglyceride, total cholesterols, and HDL-cholesterol concentration were appreciably (*P*<0.01) improved to reimburse the CCl_4_ insult.

**Table 2:** Effect of various fractions of *Launaea procumbens* on serum level of triglycerides, total cholesterol, LDL cholesterol, and HDL cholesterol in rat

Treatment	Triglycerides (mg/dl)	Total cholesterol (mg/dl)	High-density lipoprotein (mg/dl)	Low-density lipoprotein (mg/dl)
Control	12.3±1.35†	6.1±0.25†	8.6±1.71†	4.8±0.82†
DMSO+olive oil	13.26±2.50†	6.4±0.22†	9.5±1.20†	5.18±0.72†
3 ml/kg CCl_4_	21.53±1.58**	11.2±0.23**	5.8±2.18**	9.8±0.67**
100 mg/kg LPEE+CCl_4_	16.4±1.8*†	7.0±0.39**†	7.41±1.9†	7.3±0.95**†
200 mg/kg LPEE+CCl_4_	14.7±2.09†	6.5±0.62†	7.94±0.92†	6.0±0.59†
200 mg/kg LPEE alone	12.4±3.21†	5.8±0.42†	8.04±0.76†	5.1±0.15†

Mean±SE (*n*=6 number).

*, ** Significance from the control group at *P<*0.05 and *P<*0.01 probability levels.

† Significance from the CCl_4_ group at *P<*0.01 probability level.

### Indices of hepatotoxicity: serum markers

The expression levels of serum makers, namely ALT, AST, ALP, γ-GT, CYP 2E1, and p53, are susceptible to hepatotoxin and are markers of liver injury and oxidative stress, which promote the release of aminotransferase from hepatocytes into the blood stream. The marked protective effects of LEA on serum marker are shown in Table 3. Induction of CCl_4_ significantly increases (*P*<0.01) the activity of liver serum marker enzymes (ALT, AST, ALP, γ-GT) while decreasing (*P<*0.01) the expression level of CYP 2E1 and p53. The secretion of these enzymes and expression of CYP 2E1 and p53 was significantly improved (*P*<0.01) by 100 mg/kg and 200 mg/kg b.w. LEA comparatively to control rat viewing that LEA is possessed bioactive hepatoprotectant compounds however marked protection was noted with 200 mg/kg b.w. LEA.

**Table 3:** Effect of ethyl acetate fraction of *Launaea procumbens* on liver function of rat

Treatment	ALT (U/L)	AST (U/L)	ALP (U/L)	γ-GT (nM/min/mg protein)	P53	CYP 2E1
Control	32.17±2.12†	83.83±2.74†	248.00±3.93†	70.50±2.23†	48.3±2.38†	28.0±2.8†
DMSO+olive oil	32.50±2.05†	84.67±2.75†	249.67±3.68†	71.33±2.04†	49±2.03†	29±5.0†
3 ml/kg CCl_4_	91.33±3.42**	228.00±4.27**	505.33±6.49**	119.33±3.12**	27.6±2.46**	17.1±2.0**
100 mg/kg LPEE+CCl_4_	77.67±3.36**†	140.83±3.24**†	388.33±3.83**†	106.50±3.77**†	53±3.78**†	28±1.2**†
200 mg/kg LPEE+CCl_4_	38.17±2.77†	102.17±3.96**†	262.00±2.37†	78.33±3.30†	57±2.63*†	30±2.3*†
200 mg/kg LPEE alone	35.67±3.01†	98.15±2.12†	248.12±2.89†	71.43±5.10†	49±1.61†	29±3.0†

Mean±SE (*n*=6 number).

*, ** Significance from the control group at *P<*0.05 and *P<*0.01 probability levels.

† Significance from the CCl_4_ group at *P<*0.01 probability level.

### CCl_4_ induction and antioxidant status in rat liver

Antioxidant enzymes system detoxifies ROS and maintains cellular balance. Treatment of CCl_4_ significantly decreased (*P*<0.01) the activity of CAT, SOD, GST, GSH-Px, GSR, and GSH while increasing the TBARS content in rat liver. Co-treatment of 100 mg/kg and 200 mg/kg b.w. LEA in rat liver markedly improved (*P*<0.01) the reduction in the activities of antioxidant, and phase II metabolizing enzymes dose dependently justified that LEA could be used as antioxidant in daily diet (Table 4).

**Table 4:** Effect of ethyl acetate fraction of *Launaea procumbens* on liver antioxidant profile

Treatment	CAT (U/min)	SOD (U/mg protein)	GST (nM/min/mg protein)	GSH-Px (nM/min/mg protein)	GSH (M/g tissue)	TBARS (nM/min/mg protein)	GSR (nM/min/mg protein)
Control	4.397±0.275†	24.0±2.27†	128.50±4.62†	77.50±3.38†	0.738±0.0201†	78.67±6.56†	147.33±6.01†
DMSO+olive oil	4.242±0.407†	23.0±2.34†	126.67±4.21†	77.0±3.10†	0.708±0.0105†	79.00±7.45†	145.33±6.23†
3 ml/kg CCl_4_	2.590±0.240**	13.50±1.34**	68.83±4.57**	51.83±2.89**	0.236±0.0066**	158.83±8.57**	88.00±3.61**
50 mg/kg Rutin+CCl_4_	4.000±0.163†	21.0±1.83†	121.83±3.57†	71.8±2.39†	0.728±0.0432†	87.27±5.23†	142.17±6.22†
100 mg/kg LPEE+CCl_4_	3.7±0.25**†	18.667±0.9**†	97.17±4.09**†	67.5±2.4*†	0.550±0.0123**†	109.50±3.81**†	107.5±3.3**†
200 mg/kg LPEE+CCl_4_	3.9183±0.06†	22.17±1.14†	122.17±3.24†	70.7±1.62†	0.655±0.0118†	94.00±4.50†	132.33±2.89†
200 mg/kg LPEE alone	4.63±0.115†	26.0±2.38†	131.00±4.12†	78.8±3.30†	0.740±0.0101†	78.0±3.51†	150.50±6.38†

Mean±SE (*n*=6 number).

*, ** Significance from the control group at *P<*0.05 and *P<*0.01 probability levels.

† Significance from the CCl_4_ group at *P<*0.01 probability level.

### Effect of LEA on DNA damages (ladder assay, DPA assay)

**Fig. 1:**
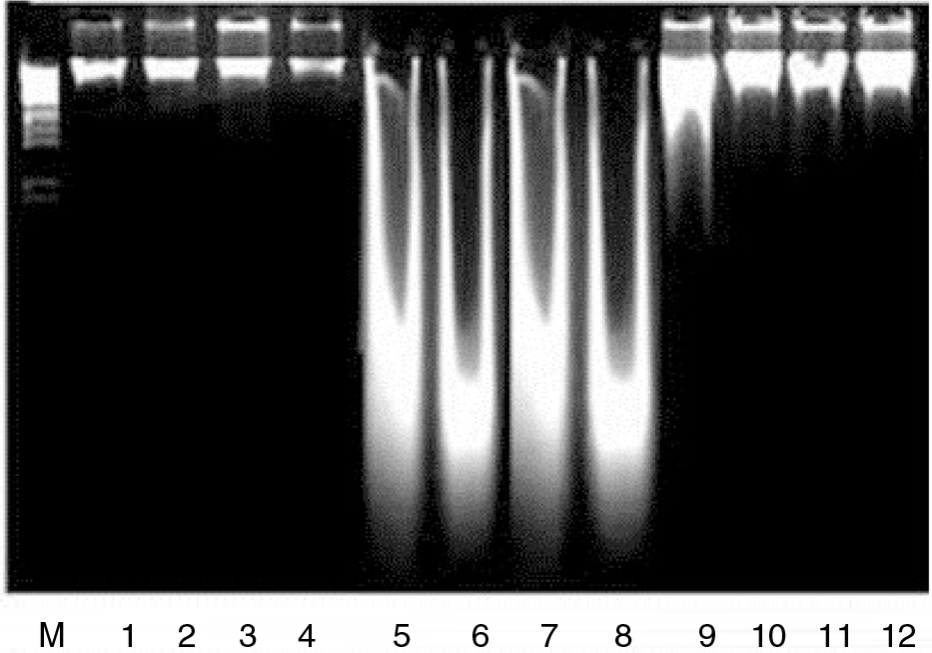
The protective effects of extract on DNA. Lanes 1–4, non-treated control; 5–8, CCl_4_ treated rats; 9–10, CCl_4_+100 mg/kg b.w. LEA; 11–12, CCl_4_+200 mg/kg b.w. LEA.

CCl_4_ free radicals fuse with DNA forming adduct and induce damages/mutation in the liver DNA of rats. The effects of LEA against CCl_4_ toxicities on DNA damages are shown in Fig. 1. CCl_4_ administration significantly increased the DNA damage which was significantly improved (*P*<0.01) by LEA depending on the dose amount as shown by band pattern and quantification (DPA assay) of different groups, when compared with the CCl_4_ group (Table 1).

## Discussion

ROS are extremely reactive molecules, resulting from the metabolism of oxygen. These ROS can cause widespread damage to cells and tissues; causes degenerative disorders, such as cardiovascular disease, oxidative stress, aging; and causes neurodegenerative diseases, such as Alzheimer's disease, mutations, and cancer (32, 33). Free radicals induced from CCl_4_ during metabolism cause liver, lung, and kidney injuries in experimental animals such as rats (10). CCl_4_ is metabolized by cytochrome P450 into trichloromethyl (CCl_3_*) and peroxy trichloromethyl (*OOCCl_3_) radicals leading to the accumulation of lipid peroxidation products that cause renal and hepatic injuries (6, 34).

Medicinal plants composed of different amounts of antioxidants play the main role in controlling various pathological conditions, including oxidative stress, cancer, cardiovascular diseases, liver diseases, and lipid peroxidation (35, 36). ROS generated due to CCl_4_ administration are more reactive and toxic than the parent compound. Metabolism of CCl_4_ occurs in the endoplasmic reticulum and the isoenzyme concerned in this process is CYP 2E1 (37) and elevation of ALT, AST, ALP, and GGT in serum (38, 39). Our results showed that an active free radical of CCl_4_ caused reduction of CYP 2E1, which was markedly improved by oral supplementation of LEA. Our result was similar to that of a previous investigation which found that polyphenolic natural bioactive compounds are responsible for its fortification (40). Our result coincides with other studies (41). Cholesterol profile is an important marker of free radical–induced toxicity. Maintenance of increased concentration of serum LDH, TG, total cholesterol, and LDL, and decreased HDL at near-normal values with co-treatment of various concentrations of ethyl acetate fraction demonstrated the hepatoprotective effect of *L. procumbens*. Similar investigations were reported by Lin et al. (41), while working on hepatoprotective effects of bioactive compounds of plants against carbon tetrachloride–induced hepatic injury in rats.

CCl_4_ free radicals cause the peroxidation of the polyenoic lipids of the endoplasmic reticulum and decrease the activities of antioxidant enzymes (3, 42, 43). Co-administration of LEA markedly erased the toxicity of CCl_4_ and the enzymatic activities of antioxidant enzymes toward the normal range in this experiment. A similar result has been documented in various studies (44, 45). CCl_4_ induces lipid peroxidation and increases the TBARS contents in liver cells. TBARS is a major reactive aldehyde occurring during the peroxidation of polyunsaturated fatty acids (PUFA), a useful indicator showing tissue damages including a series of chain reactions (46). Administration of LEA significantly recovered the TBARS content near to control rats as was revealed by other plant extracts (3, 9). Free radicals–induced lipid peroxide react with DNA to form the adduct M1G, the mutagenic pirimedopurinone adduct of deoxyguanosine (47), as was revealed by DNA ladder assay (Fig. 1). Administration of LEA improved the DNA fragmentation, which is in close agreement with other studies (9) (Khan et al., 2009). DNA damage causes expression of p53, blocks cell cycle, and repairs their DNA damage (15).

## Conclusion

Ethyl acetate fraction of *L. procumbens* regulated the activities of serum markers, antioxidant enzymes, CYP 2E1, and p53 protein because of the presence of bioactive constituents. It is therefore suggested that we isolate and purify these compounds to be used in future as a drug against oxidative stress and liver carcinoma.
